# Autoimmune and lymphoproliferative outcomes after Kawasaki disease: a 20-year population-based cohort study

**DOI:** 10.1007/s00431-026-07356-w

**Published:** 2026-09-03

**Authors:** Rim Kasem Ali Sliman, Nadin Shalaby, Hilla Cohen, Dana Hadar, Inbal Halabi, Mohamad Hamad Saied

**Affiliations:** 1 The Ruth and Bruce Rappaport Faculty of Medicine, Technion–Israel Institute of Technology, Haifa, Israel; 2 Department of Pediatrics, Clalit Health Services, Carmel Medical Center, 7 Michal St., 3436212 Haifa, Israel; 3 Research Authority, Clalit Health Services, Carmel Medical Center, Haifa, Israel; 4 Department of Pediatric Immunology and Infectious Diseases, Wilhelmina Children’s Hospital, University Medical Center Utrecht, Utrecht, the Netherlands

**Keywords:** Kawasaki disease, Autoimmune disease, Hodgkin lymphoma, Psoriasis, Vitiligo, Long-term outcomes, Cohort study

## Abstract

Kawasaki disease (KD) is an acute systemic vasculitis of childhood associated with profound immune dysregulation that may persist beyond the acute inflammatory phase. The long-term risks of autoimmune diseases and lymphoproliferative malignancies in children with KD remain incompletely defined. To determine whether children with KD carry increased 20-year risks of autoimmune and lymphoproliferative outcomes compared with matched controls. We conducted a retrospective matched cohort study using the Clalit Health Services database (2002–2022). Children with KD (*n* = 2,126) were matched 1:5 to controls (*n* = 10,630) by sex and birthdate (± 30 days) and followed through December 2024. Outcomes comprised psoriasis, vitiligo, hypothyroidism, type 1 diabetes mellitus (T1DM), celiac disease, inflammatory bowel disease (IBD), immune thrombocytopenia (ITP), Hodgkin lymphoma (HL), and non-Hodgkin lymphoma (NHL). Adjusted hazard ratios (AHRs) with 95% confidence intervals were estimated using Cox proportional hazards models at 2, 5, 10, 15, and 20 years. KD was associated with a significantly increased risk of psoriasis from 2 years (AHR 3.03, 95% CI 1.19–7.73) through 15 years (AHR 1.50, 95% CI 1.01–2.23), attenuating to non-significance at 20 years. Vitiligo reached statistical significance at 10 years (AHR 1.38, 95% CI 1.03–1.86). A nominally significant elevation in HL risk was observed from 15 years onward (AHR 14.99, 95% CI 1.55–145.12, at 15 years; AHR 7.95, 95% CI 1.32–48.00, at 20 years); however, these estimates are based on three events in the KD group versus one to two in controls, yielding confidence intervals spanning nearly two orders of magnitude, and must be interpreted as exploratory signals only. No significant associations were found for hypothyroidism, T1DM, ITP, celiac disease, IBD, or NHL.

*Conclusion*: KD was associated with a sustained increase in psoriasis risk and a consistent trend toward increased vitiligo risk, reaching statistical significance at 10 years. An exploratory HL signal emerged during long-term follow-up but was based on very few events. Overall, these findings support selective long-term immune dysregulation following KD and highlight the importance of dermatologic awareness during long-term follow-up.

**Table:** 

**What is known:**
• *Kawasaki disease (KD) causes profound immune dysregulation that may persist beyond the acute phase.*
• *Whether KD increases long-term risks of autoimmune and lymphoproliferative disease remains unclear.*
**What is new:**
• *Over 20 years, KD was associated with sustained psoriasis risk and a significant rise in vitiligo risk at 10 years.*
• *No increased risk was found for hypothyroidism, type 1 diabetes, celiac disease, IBD, or ITP, indicating selective rather than generalized immune dysregulation.*

**Supplementary Information:**

The online version contains supplementary material available at https://doi.org/10.1007/s00431-026-07356-w.

## Introduction

Among childhood vasculitides, Kawasaki disease (KD) is distinguished by its predilection for children under five years of age, its unclear etiology, and its potential to cause lasting coronary artery injury. Recognized as the leading cause of acquired cardiac disease in children in high-income countries, KD is characterized by profound immune activation whose effects may extend well beyond the acute inflammatory phase [1, 2]. Incidence varies markedly by ethnicity, with the highest rates reported in Japan (approximately 300 per 100,000 children under five years) and lower but rising rates in Western populations [1, 2]. Diagnosis remains clinically based, as no pathognomonic laboratory test exists, and incomplete presentations continue to pose diagnostic challenges that may lead to delayed recognition or misclassification [1, 3].

Intravenous immunoglobulin combined with high-dose aspirin has substantially reduced acute coronary complications [1, 4], yet the underlying immunopathogenesis remains incompletely understood. Current evidence supports a model in which an environmental or infectious trigger elicits an aberrant immune response in genetically predisposed children, characterized by dysregulation of both innate and adaptive immunity, T- and B-cell hyperactivation, cytokine storm, and systemic inflammation [4–6]. Critically, these immune aberrations have been documented to persist well beyond the acute phase, in some cases for months to years [4–6]. Recent work has further suggested that KD may induce trained immunity, a lasting functional reprogramming of innate immune cells through epigenetic and metabolic modifications. However, this remains a hypothetical framework rather than an established mechanism underlying the long-term immune consequences of KD [7, 8].

Epidemiological evidence increasingly suggests an elevated long-term autoimmune risk following KD. A recent national cohort study of 16,398 Korean children with 12 years of follow-up reported associations between KD and several immune-mediated outcomes, including vitiligo and immune thrombocytopenia, although no significant association with psoriasis was identified in that cohort [9]. A large Canadian population-based cohort study similarly demonstrated an increased incidence of chronic immune-mediated inflammatory diseases following KD [10]. In addition, a Danish registry study of 820 children demonstrated a 2.42-fold elevated cancer risk over 30 years of follow-up [11], and a systematic review identified 25 cases of lymphoid neoplasms diagnosed in close temporal proximity to a KD episode [12]. Despite this growing body of evidence, comprehensive long-term assessments that simultaneously evaluate autoimmune diseases and lymphoproliferative malignancies, with adequate sample size and follow-up duration, remain limited [3, 11].

The primary objective of this study was to determine whether children with KD carry increased 20-year risks of a broad spectrum of autoimmune diseases and lymphoproliferative conditions compared with matched controls, using a large national healthcare database with near-complete diagnostic capture.

## Methods

### Study design and population

This retrospective matched cohort study used data from the CHS database, covering January 1, 2002, to December 31, 2022. CHS is Israel’s largest health maintenance organization, insuring approximately 4.7 million members, about 52% of the Israeli population, with comprehensive longitudinal electronic health records. CHS is a non-profit health fund operating under Israel’s National Health Insurance Law, under which funds are reimbursed through age- and risk-adjusted capitation rather than fee-for-service diagnostic billing. Diagnoses are recorded for clinical care and carry no reimbursement incentive for over-coding, which reduces the likelihood of systematic over-reporting. Children diagnosed with KD were matched 1:5 to general pediatric controls by sex and birthdate (± 30 days). This is a matched cohort study with incidence-density sampling, and controls contributed follow-up time from the shared index date. A 1:5 matching ratio was selected to increase statistical precision while retaining feasibility. Controls were selected from the general CHS-insured pediatric population and assigned the index date of their matched KD case; Matching on sex and birthdate was performed to account for the well-established demographic determinants of both KD incidence and autoimmune disease risk. Participants with documented pre-existing conditions relevant to a given outcome were excluded from the corresponding outcome analysis. All participants were followed through December 31, 2024. Each child’s record was linked to the corresponding maternal and paternal records, which contained data on the presence or absence of the autoimmune-related conditions under study (Supplementary Table 1).

### Eligibility criteria

The cases were children under 18 years of age at the time of KD diagnosis who met the American Heart Association 2017 diagnostic criteria [1]. Controls had no KD diagnosis and no prior record of the specific outcome under analysis. Participants with documented pre-existing relevant conditions before the index date were excluded from the respective outcome analyses, as detailed in Supplementary Table 1.

### Outcome measures

Primary outcomes were incident diagnoses of psoriasis, vitiligo, ITP, HL, NHL, hypothyroidism, T1DM, celiac disease, and IBD, identified using predefined ICD-9 and ICD-10 diagnostic codes recorded in the CHS electronic health record. Hypothyroidism was defined as physician-recorded primary hypothyroidism, including Hashimoto thyroiditis and both clinical and subclinical hypothyroidism. Outcomes were pre-specified and restricted to selected immune-mediated and lymphoproliferative conditions with a previously reported or biologically plausible association with Kawasaki disease and reliable administrative coding. Outcome assessments were pre-specified at 2, 5, 10, 15, and 20 years after the index date.

### Statistical analysis

Baseline characteristics were compared between the KD and control groups using the chi-square test for categorical variables and the independent-samples t-test for continuous variables. Incidence rates were calculated per 100,000 person-years for all outcomes at each pre-specified follow-up interval. Time-to-event analyses were performed using Cox proportional hazards regression, yielding adjusted hazard ratios (AHRs) with 95% confidence intervals (CIs). Models were adjusted for sex, age at the index date, and documented parental (maternal and paternal) autoimmune disease, selected a priori based on their established association with autoimmune disease risk and their availability within the CHS database. Cumulative incidence functions were estimated using the Kaplan–Meier method and compared between groups with the log-rank test. The proportional hazards assumption was evaluated using scaled Schoenfeld residuals. No adjustment for multiple comparisons was applied, given the strong biological rationale underpinning each tested association. All analyses were conducted in R version 4.1.2 (R Foundation for Statistical Computing, Vienna, Austria), with survival analyses performed using the survival package. A two-sided significance threshold of α = 0.05 was applied throughout.

### Ethical considerations

The study was approved by the CHS Institutional Review Board (Helsinki Committee; approval number: CMC-0070–24). Individual informed consent was waived owing to the retrospective design and the use of de-identified data, in accordance with national regulations.

## Results

**Fig. 1:**
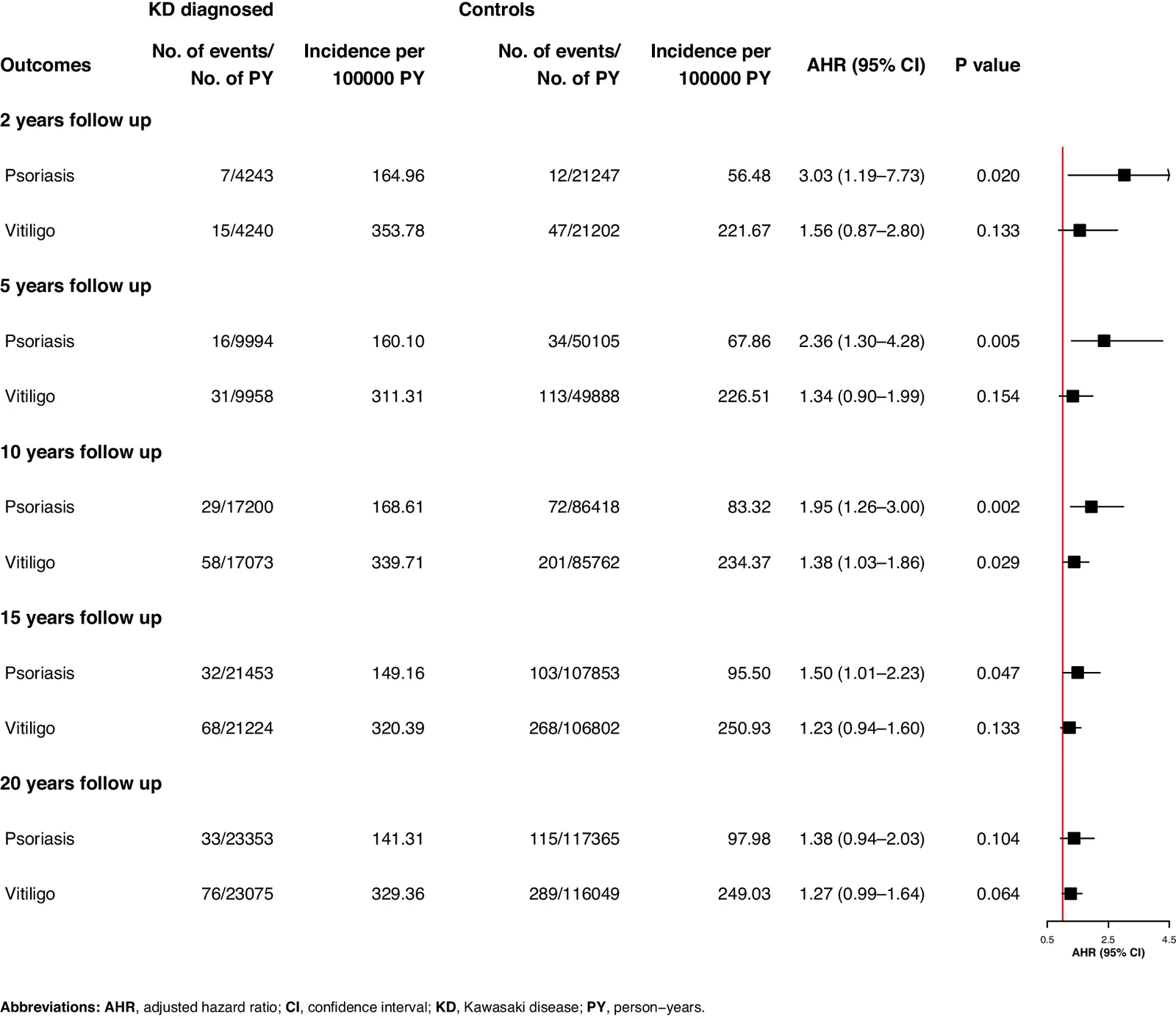
Risk of dermatologic autoimmune outcomes (psoriasis and vitiligo) in children with Kawasaki disease (KD) versus matched controls at 2, 5, 10, 15, and 20 years of follow-up. For each outcome and time point, the number of events/person-years, incidence per 100,000 person-years, adjusted hazard ratio (AHR) with 95% confidence interval (CI) from Cox proportional hazards regression, and P value are shown; AHRs are plotted on the right

**Fig. 2:**
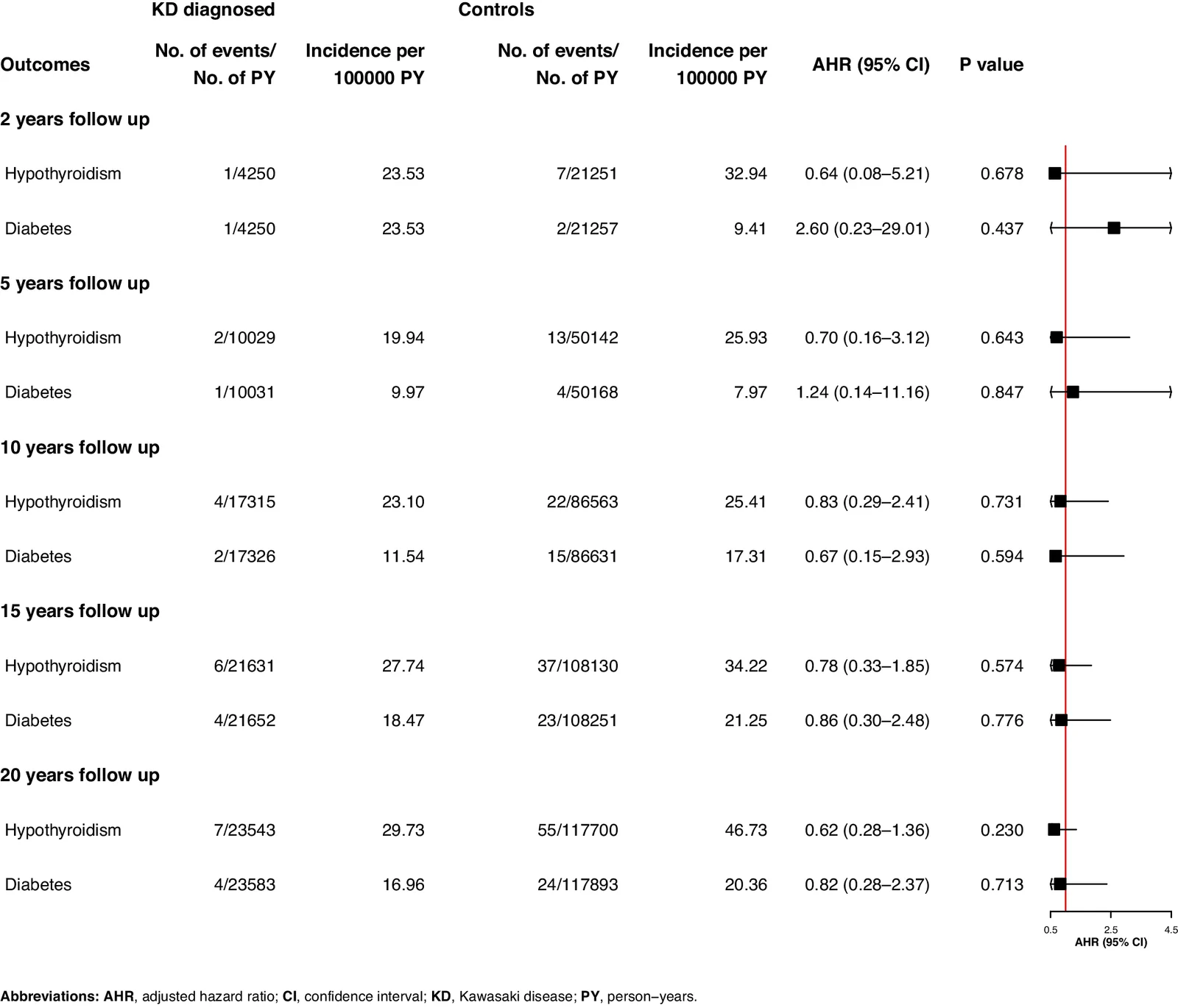
Risk of endocrine autoimmune outcomes (hypothyroidism and type 1 diabetes mellitus) in children with KD versus matched controls at 2, 5, 10, 15, and 20 years of follow-up; columns and plotted estimates as in Fig. 1

**Fig. 3:**
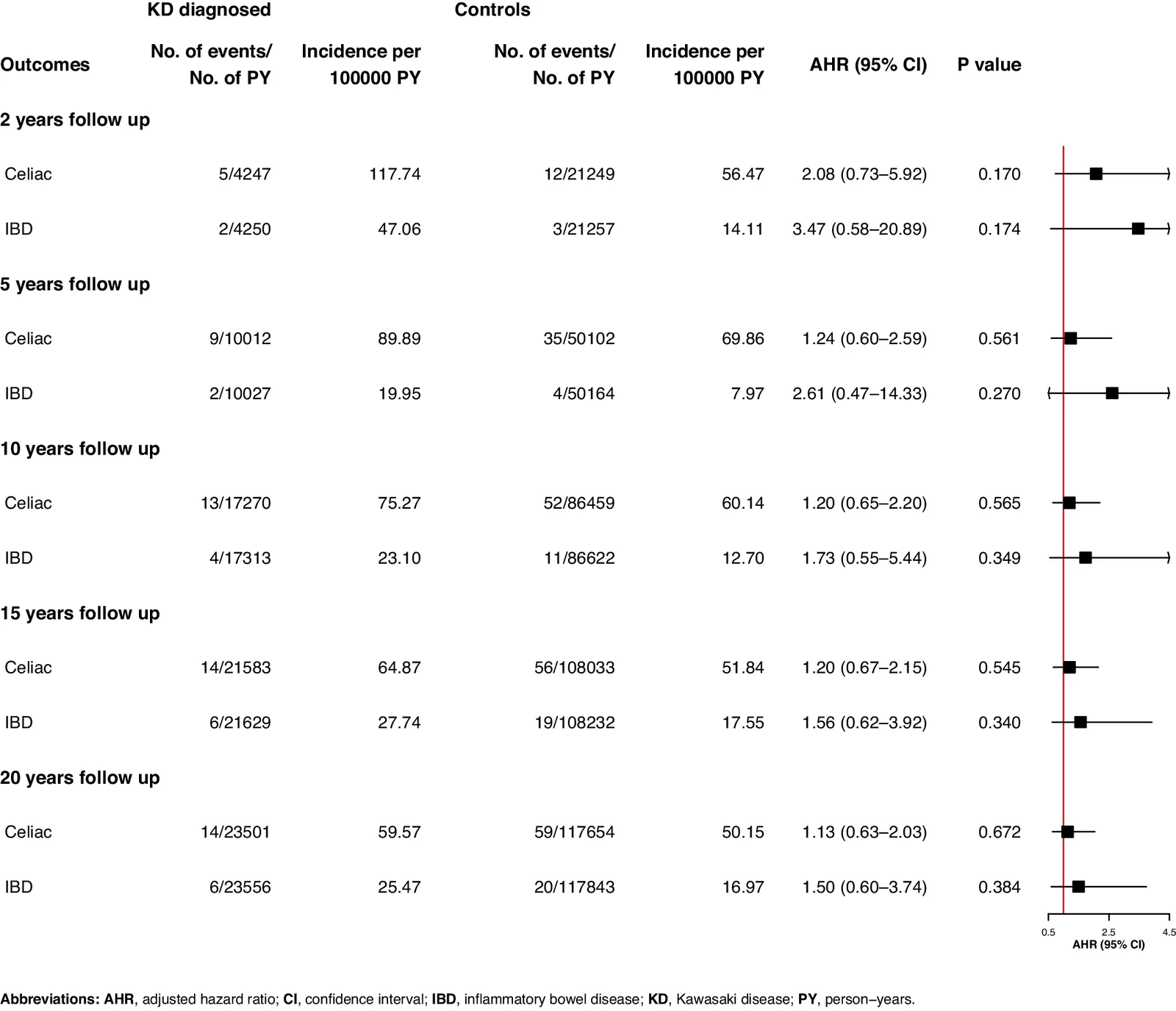
Risk of gastrointestinal autoimmune outcomes (celiac disease and inflammatory bowel disease) in children with KD versus matched controls at the five pre-specified time points; columns and plotted estimates as in Fig. 1

**Fig. 4:**
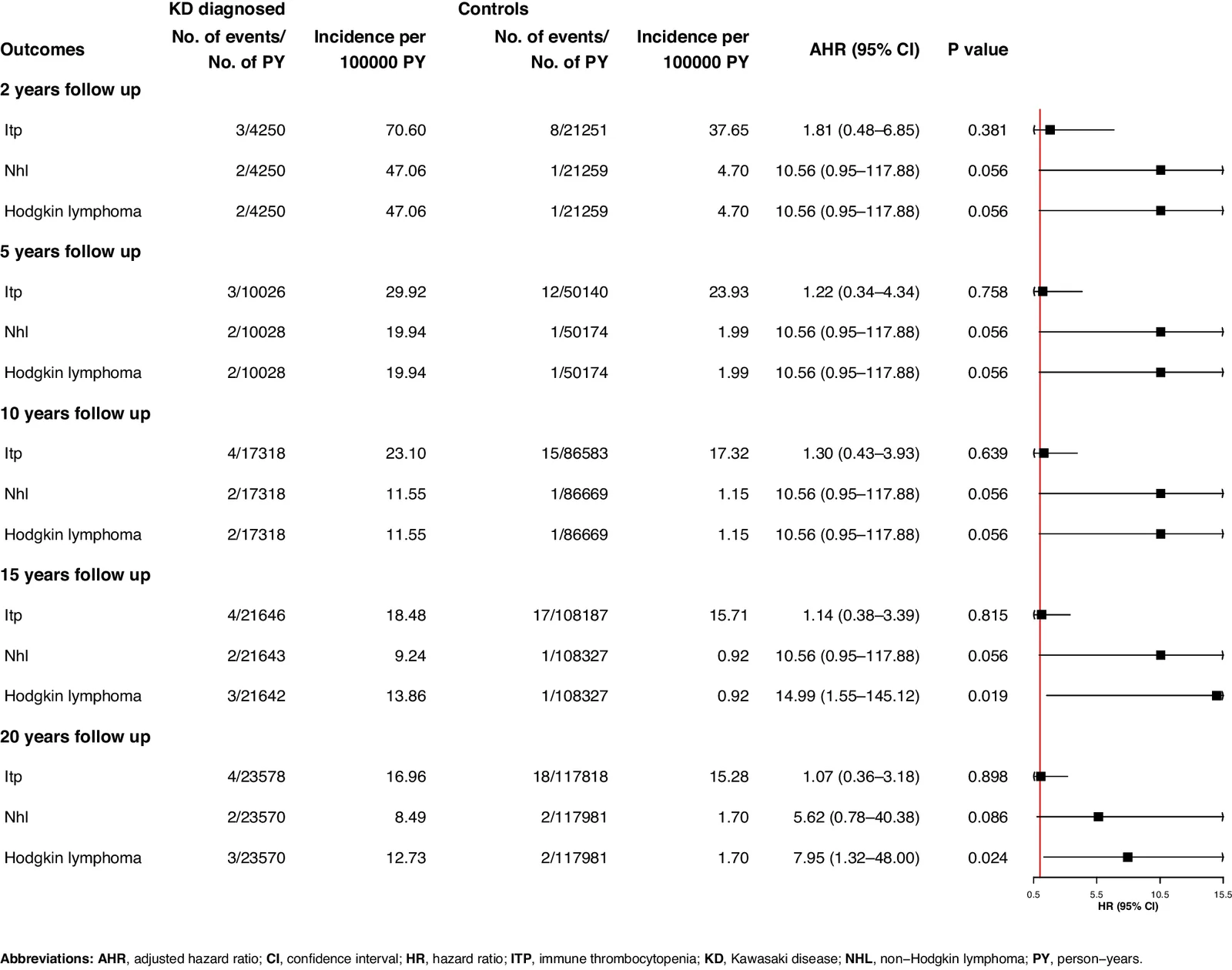
Risk of hematologic and lymphoproliferative outcomes (immune thrombocytopenia, non-Hodgkin lymphoma, and Hodgkin lymphoma) in children with KD versus matched controls at the five pre-specified time points; columns and plotted estimates as in Fig. 1

The cohort comprised 2,126 children with KD and 10,630 matched controls. Groups were well balanced, with a mean age at the index date of 3.07 ± 2.90 years in the KD group and 3.08 ± 2.90 years in controls, and males accounted for 62% of each group (Table 1). Cumulative incidence estimates and incidence rates per 100,000 person-years for all outcomes are presented in Figs. 1, 2, 3, 4, allowing interpretation of both the relative and absolute risks.

**Table 1:** Baseline characteristics of children with Kawasaki disease and matched controls

Characteristic	Kawasaki Disease *N* = 2,126	Control, *N* = 10,630	*P*-value
Age_at_index_date Mean (SD)	3.07 (2.90)	3.08 (2.90)	> 0.9
Gender Males *n* (%)	1,327(62%)	6,635(62%)	> 0.9

Among dermatologic outcomes, psoriasis demonstrated a significant and sustained increase in risk among children with KD: AHR 3.03 (95% CI 1.19–7.73, *p* = 0.020) at 2 years, 2.36 (95% CI 1.30–4.28, *p* = 0.005) at 5 years, 1.95 (95% CI 1.26–3.00, *p* = 0.002) at 10 years, and 1.50 (95% CI 1.01–2.23, *p* = 0.047) at 15 years, attenuating to non-significance at 20 years (AHR 1.38, 95% CI 0.94–2.03, *p* = 0.104) (Fig. 1; Supplementary Fig. 1). Vitiligo reached statistical significance only at 10 years (AHR 1.38, 95% CI 1.03–1.86, *p* = 0.029), with a borderline non-significant trend persisting at 20 years (AHR 1.27, 95% CI 0.99–1.64, *p* = 0.064) (Fig. 1; Supplementary Fig. 2).

No significant associations were observed for endocrine outcomes at any time point. AHRs for hypothyroidism ranged from 0.64 (95% CI 0.08–5.21) at 2 years to 0.62 (95% CI 0.28–1.36) at 20 years (Fig. 2; Supplementary Fig. 3), and AHRs for T1DM ranged from 2.60 (95% CI 0.23–29.01) at 2 years to 0.82 (95% CI 0.28–2.37) at 20 years (Fig. 2; Supplementary Fig. 4). Similarly, no significant associations were found for gastrointestinal outcomes. AHRs for celiac disease declined from 2.08 (95% CI 0.73–5.92) at 2 years to 1.13 (95% CI 0.63–2.03) at 20 years (Fig. 3; Supplementary Fig. 5), and AHRs for IBD ranged from 3.47 (95% CI 0.58–20.89) at 2 years to 1.50 (95% CI 0.60–3.74) at 20 years (Fig. 3; Supplementary Fig. 6).

Among hematologic and lymphoproliferative outcomes, ITP was numerically more frequent among children with KD at all time points but did not reach statistical significance at any interval (AHR 1.81, 95% CI 0.48–6.85 at 2 years; AHR 1.07, 95% CI 0.36–3.18 at 20 years) (Fig. 4; Supplementary Fig. 7). For NHL, the point estimate was markedly elevated but remained statistically non-significant through 15 years of follow-up (AHR 10.56, 95% CI 0.95–117.88, *p* = 0.056, at each of 2, 5, 10, and 15 years), attenuating at 20 years (AHR 5.62, 95% CI 0.78–40.38, *p* = 0.086) (Supplementary Fig. 8). These estimates were based on two events in the KD group and one to two events in the control group across the entire follow-up period and should therefore be interpreted with considerable caution.

For HL, the point estimate was similarly elevated but remained non-significant through 10 years (AHR 10.56, 95% CI 0.95–117.88, *p* = 0.056 at 2, 5, and 10 years), reaching nominal statistical significance from 15 years onward: AHR 14.99 (95% CI 1.55–145.12, *p* = 0.019) at 15 years and AHR 7.95 (95% CI 1.32–48.00, *p* = 0.024) at 20 years (Fig. 4; Supplementary Fig. 9). However, these estimates were based on only three HL events in the KD group versus one event in the control group at 15 years, and three versus two events at 20 years. The resulting confidence intervals were wide, reflecting substantial statistical imprecision, and the nominal statistical significance should therefore be interpreted as exploratory rather than confirmatory. These absolute event counts highlight the limited number of events underlying the hazard ratio estimates and should be considered when interpreting these findings.

## Discussion

In this large population-based cohort study of children with KD followed for up to 20 years, we identified a consistent and significant elevation in psoriasis risk from 2 through 15 years after diagnosis, and a significant but temporally isolated elevation in vitiligo risk at 10 years. A nominally significant signal for HL emerged only from 15 years onward but was based on very few events and should therefore be regarded as exploratory. No significant associations were observed for hypothyroidism, T1DM, ITP, celiac disease, IBD, or NHL. The divergent temporal patterns across outcomes, with early and sustained onset for psoriasis, a single significant time point for vitiligo, and a late and imprecisely estimated HL signal, suggest that different biological mechanisms may underlie these associations. Early manifestations may reflect acute immune activation and inflammatory priming, whereas delayed outcomes may reflect chronic immune dysregulation, epigenetic modifications, or cumulative immune injury that evolves over time.

The significant and sustained association with psoriasis complements prior evidence from case reports and small series. Eberhard et al. identified psoriasiform eruptions in 1.9% of 476 children with KD over a 10-year follow-up period [13], and several subsequent reports have documented the immunological overlap between KD and psoriasis [14–16]. Our data extend these observations by demonstrating peak risk during the first two years after KD, with significant elevation persisting through 15 years and attenuating to non-significance at 20 years. The earliest estimate, at two years of follow-up (median age approximately five years), was based on only seven events. Early-childhood psoriasis is uncommon but recognized, and closer medical follow-up after KD may have contributed to earlier detection. The associations observed at 5 and 10 years are more robust and less likely to reflect chance or surveillance bias. This temporal pattern is consistent with an early immunological window in which KD-related dysregulation of the IL-23/Th17 axis and the Koebner phenomenon most directly precipitate psoriasis in genetically predisposed individuals [4], followed by gradual waning of the acute immune stimulus and increasing influence of other genetic and environmental determinants with advancing age. Notably, our findings differ from the Korean national cohort study by Kwak et al., which did not identify a significant KD–psoriasis association [9], possibly because of differences in study populations, healthcare systems, and exclusion of early post-KD cases, precisely the period of highest observed risk in our dataset. Similarly, the Canadian cohort reported by Fung et al. did not show a statistically significant association with psoriasis, potentially owing to a shorter follow-up period [10]. These discrepancies underscore the importance of extended observation windows and complete capture of early post-KD events in detecting associations that shorter studies may miss.

The vitiligo finding warrants cautious interpretation. Statistical significance was reached at only one of five pre-specified time points (10 years, AHR 1.38, 95% CI 1.03–1.86), with the estimates at 2, 5, 15, and 20 years not reaching significance, though the 20-year estimate was borderline (AHR 1.27, 95% CI 0.99–1.64, *p* = 0.064). Given the evaluation of multiple pre-specified outcomes across several follow-up intervals, this isolated statistically significant finding should be interpreted cautiously and regarded as exploratory pending confirmation in independent cohorts. Nonetheless, the direction of the association was consistent across all time points, the 10-year estimate is biologically plausible, and the finding accords with the large Korean national cohort by Kwak et al., which provided population-level evidence linking KD to vitiligo [9], and with the case report by Cho et al. of histologically confirmed vitiligo developing in areas previously affected by KD rashes [17]. If real, the delayed onset would suggest that KD may initiate a chronic autoimmune process with progressive melanocyte destruction mediated by T-cell cytotoxicity and IFN-γ-driven chemotaxis [4, 18], rather than a transient cutaneous reaction. A plausible framework for such delayed effects is trained immunity, whereby the intense inflammatory insult of KD induces lasting epigenetic and metabolic reprogramming of innate immune cells, including histone modifications at promoters of inflammatory response genes and a shift toward aerobic glycolysis, which may extend to hematopoietic stem and progenitor cells in the bone marrow, sustaining a chronic pro-inflammatory state for years after the acute event [7, 8]. Trained immunity is proposed here only as a hypothetical framework. The present study is epidemiological and provides no mechanistic evidence; therefore, this hypothesis requires validation in dedicated experimental and translational studies.

Chronic autoimmune inflammation differs fundamentally from the acute, self-limited inflammatory course of KD, and mechanisms proposed for lymphoma development in chronic autoimmune diseases cannot be directly extrapolated to KD. The nominally significant HL findings at 15 and 20 years warrant careful contextualization before any clinical interpretation. The statistically significant AHRs of 14.99 (95% CI 1.55–145.12) at 15 years and 7.95 (95% CI 1.32–48.00) at 20 years are each derived from three HL events in the KD group versus one and two events in the control group, respectively. The resulting confidence intervals were extremely wide, reflecting substantial statistical imprecision, and the nominal p-values below 0.05 should not be interpreted as robust evidence of a causal association. These results are best understood as exploratory signals that require replication in larger, independent cohorts before any firm conclusions can be drawn. With that important caveat stated, the direction of these estimates is biologically plausible and consistent with prior observations. The Danish registry study by Nielsen et al. demonstrated a 2.42-fold increase in long-term cancer risk following KD [11], and Yu et al. reported increased lymphoma risk among patients with systemic inflammatory autoimmune diseases [19]. Unlike the studies by Suzuki et al. [20] and Banday et al. [12], which focused on lymphomas occurring in close temporal proximity to acute KD (weeks to months), the present data suggest that any such association, if real, may persist well into adolescence and young adulthood. Proposed biological mechanisms include cytokine-driven clonal expansion of lymphocytes and long-term impairment of immune surveillance, potentially facilitating neoplastic escape [19–21]. The late emergence of the HL signal, coinciding with the adolescent and young adult age period when HL incidence naturally peaks in the general population, raises the additional question of whether KD-related immune dysregulation might interact with the physiological immune maturation of this developmental stage. These are questions for future, adequately powered research. From a clinical standpoint, the present data do not support routine oncologic surveillance in this population. Still, they do suggest that clinicians maintaining long-term follow-up should remain alert to hematologic symptoms in this population.

Equally informative are the null findings. Over 20 years of follow-up, no significant associations were observed for celiac disease, IBD, hypothyroidism, T1DM, or ITP. These findings align with the Canadian cohort reported by Fung et al. [10], which similarly found no increased risk of IBD or T1DM following KD, and suggest that despite the marked immune activation characteristic of acute KD, the disease does not broadly predispose to a wide spectrum of immune-mediated disorders. Our findings differ from those of Stagi et al. [22], who reported a higher prevalence of celiac disease following KD, and from Kwak et al. [9], who identified an increased risk of ITP. These discrepancies may reflect ethnic and genetic variability, differences in healthcare systems and diagnostic capture, or limited statistical power related to the rarity of several outcomes during long-term follow-up. Taken together, the selective elevation in risk observed for psoriasis and vitiligo, alongside the absence of associations with endocrine and gastrointestinal autoimmune diseases, supports the concept that KD-related immune dysregulation is pathway-specific rather than a manifestation of generalized immune tolerance failure. Although the IL-23/Th17 axis has been implicated in several immune-mediated diseases, activation of a shared immunological pathway does not necessarily result in the same clinical phenotype. Consistent with this, we observed no increased risk of IBD, while spondyloarthropathies were beyond the scope of the present study. Limited statistical power for rarer outcomes should also be considered.

Unlike systemic lupus erythematosus, in which broad tolerance failure predisposes to multi-organ autoimmunity, the immune perturbation following KD appears to predominantly involve cutaneous immune pathways while sparing endocrine and gastrointestinal immunity. The null finding for IBD is particularly instructive, given that the gut-associated immune system shares several features with the mucocutaneous inflammation of acute KD. Yet, this overlap does not translate into a persistent predisposition to chronic intestinal inflammation. Similarly, the absence of a T1DM association is consistent with the predominantly Th17/Th1-driven pathology of KD rather than the Th2-mediated mechanisms implicated in beta-cell destruction. Collectively, these observations reinforce the concept that KD initiates immune-mediated pathology along selective immunological axes rather than through diffuse breakdown of self-tolerance.

The present study extends the existing literature by providing one of the longest follow-up durations and the broadest simultaneous assessment of autoimmune and lymphoproliferative outcomes within a single KD cohort. The consistent psoriasis signal across four of five time points, the directionally consistent but statistically imprecise vitiligo association, and the exploratory HL signal together suggest that the immune consequences of KD may extend far beyond the acute illness in a subset of patients. Prospective, multi-ethnic cohort studies incorporating biomarker assessment, treatment-response stratification, and KD severity grading are needed to determine whether these associations are reproducible and to define evidence-based long-term follow-up strategies for children with a history of KD.

### Strengths and limitations

This study benefits from a large population-based design with 20 years of follow-up, a 1:5 matched cohort structure, and access to a comprehensive national healthcare database that provides near-complete diagnostic capture. Matching controls on sex and birthdate effectively controlled for major demographic confounders, and the linkage to parental health records allowed adjustment for familial autoimmune background.

Several limitations should be acknowledged. Reliance on administrative diagnostic coding introduces potential for outcome misclassification, although the use of ICD codes within a unified healthcare system substantially mitigates this concern relative to multi-payer databases. Statistical power for rare outcomes such as HL and NHL was limited by low absolute event counts, producing wide confidence intervals that warrant cautious interpretation. The retrospective design cannot exclude residual confounding from unmeasured genetic, environmental, or healthcare utilization factors. Although all study outcomes were pre-specified based on biological plausibility, evaluating multiple outcomes across several follow-up intervals increases the possibility of type I error. Accordingly, findings based on isolated statistically significant time points or very small numbers of events should be interpreted cautiously and require independent replication. Detection bias is a particular concern for mild, chronically managed conditions such as psoriasis and vitiligo. Children with KD generally undergo more frequent medical contact, particularly during the years immediately following diagnosis, which may increase the likelihood that these conditions are recognized and recorded. This mechanism could contribute to the early psoriasis signal and cannot be excluded by the present study design. Administrative coding does not capture disease extent, distribution, or treatment. Therefore, we could not distinguish limited from extensive psoriasis, single from multiple vitiligo lesions, or treated from untreated disease, and the recorded diagnoses reflect the presence rather than the severity of disease. Because IVIG responsiveness, coronary artery involvement, disease recurrence, and acute-phase severity were unavailable, these KD-specific factors may represent sources of residual confounding and prevented analyses of potentially higher-risk subgroups. Future prospective studies incorporating these variables are warranted. A formal comparison of attrition between the KD and control groups was not performed. However, the continuous healthcare enrollment structure of the CHS database reduces the likelihood of differential loss to follow-up. Finally, the predominantly Israeli study population may limit generalizability given the well-documented ethnic heterogeneity in KD susceptibility and autoimmune disease prevalence.

## Conclusions

In this 20-year population-based cohort study of 2,126 children with KD and 10,630 matched controls, KD was significantly associated with an elevated risk of psoriasis at 2, 5, 10, and 15 years after diagnosis, attenuating to non-significance at 20 years. A significant elevation in vitiligo risk was observed at 10 years, with a consistent but non-significant directional trend across all other time points; this finding should be interpreted cautiously, given that statistical significance was reached at only one pre-specified interval. A nominally significant elevation in HL risk emerged from 15 years onward. Still, this finding was based on very sparse event counts and should be regarded as exploratory and hypothesis-generating, pending replication in larger cohorts. No significant associations were found for hypothyroidism, T1DM, celiac disease, IBD, ITP, or NHL. The temporal pattern of the psoriasis association, together with the directionally consistent vitiligo findings, supports the concept of selective long-term immune dysregulation following KD rather than generalized immune tolerance failure. These findings support clinical awareness of incident autoimmune skin disease during long-term follow-up of children with a history of KD and highlight the need for larger, prospective, multi-ethnic cohort studies to clarify the nature and magnitude of these associations.

## Supplementary Information

Below is the link to the electronic supplementary material.

Supplementary file1 **Supplementary Figure 1. **Cumulative incidence of psoriasis in children with KD (teal) versus matched controls (red) over 20 years of follow-up, estimated by the Kaplan–Meier method. Numbers at risk are shown below the plot, and the 20-year adjusted hazard ratio (95% CI) is annotated within the panel. (PNG 568 KB)

Supplementary file2 **Supplementary Figure 2. **Cumulative incidence of vitiligo, presented as in Supplementary Figure 1. (PNG 567 KB)

Supplementary file3 **Supplementary Figure 3. **Cumulative incidence of hypothyroidism, presented as in Supplementary Figure 1. (PNG 101 KB)

Supplementary file4 **Supplementary Figure 4. **Cumulative incidence of type 1 diabetes mellitus, presented as in Supplementary Figure 1. (PNG 480 KB)

Supplementary file5 **Supplementary Figure 5. **Cumulative incidence of celiac disease, presented as in Supplementary Figure 1. (PNG 503 KB)

Supplementary file6 **Supplementary Figure 6. **Cumulative incidence of inflammatory bowel disease, presented as in Supplementary Figure 1. (PNG 446 KB)

Supplementary file7 **Supplementary Figure 7. **Cumulative incidence of immune thrombocytopenia (ITP), presented as in Supplementary Figure 1. (PNG 444 KB)

Supplementary file8 **Supplementary Figure 8. **Cumulative incidence of non-Hodgkin lymphoma (NHL), presented as in Supplementary Figure 1. (PNG 445 KB)

Supplementary file9 **Supplementary Figure 9. **Cumulative incidence of Hodgkin lymphoma, presented as in Supplementary Figure 1. (PNG 470 KB)

Supplementary file10 **Supplementary Table 1. **Pre-existing conditions used as exclusion criteria for the corresponding outcome analyses. For each outcome, children with the listed diagnosis recorded before the index date were excluded from that analysis. (DOCX 15 KB)

## Data Availability

No datasets were generated or analysed during the current study.
