# Supplementary material for: Autoimmune and lymphoproliferative outcomes after Kawasaki disease: a 20-year population-based cohort study
Source: Eur J Pediatr. 2026 Sep 3;185(9):716. doi: 10.1007/s00431-026-07356-w (PMC13541939; doi:10.1007/s00431-026-07356-w)
Supplement: Supplementary file 1 [file 431_2026_7356_MOESM10_ESM.docx]

Supplementary Table 1:

**Diagnosis**

Atopic dermatitis

Allergic rhinitis

Asthma

Food allergy

Drug allergy

Angioedema

Juvenile idiopathic arthritis

Systemic lupus erythematosus

Henoch-Schönlein purpura

Hypothyroidism (including Hashimoto thyroiditis, clinical and subclinical hypothyroidism)

Type 1 diabetes mellitus

Immune thrombocytopenia

Celiac disease

Inflammatory bowel disease (Crohn's disease, ulcerative colitis)

Psoriasis

Vitiligo

Hodgkin lymphoma

Non-Hodgkin lymphoma
